# Replacement of gluteal implants by polymethyl methacrylate filler: case report

**DOI:** 10.1080/23320885.2018.1549946

**Published:** 2019-01-04

**Authors:** Roberto Chacur, Honório Sampaio Menezes, Nívea Maria Bordin da Silva Chacur, Danuza Dias Alves, Rodrigo Cadore Mafaldo, Leandro Dias Gomes, Gina Matzenbacher, Renata Bataiolli

**Affiliations:** a Leger Clinic, Rio de Janeiro, Brazil;; b Leger Clinic, São Paulo, Brazil;; c Leger Clinic, Porto Alegre, Brazil

**Keywords:** Esthetics, polymethyl methacrylate, prosthesis, plastic surgery

## Abstract

Silicone prostheses are an alternative to shape the buttock but further studies are still needed to support the effectiveness of its use. A patient sought medical attention for being dissatisfied with the glutaeal silicone prostheses inserted using subcutaneous technique four years before. The treatment adopted was prosthesis removal surgery, and subsequent filling with PMMA.

## Introduction

There is a great demand from patients looking for procedures to sculpt the body [1]. Plastic surgery, for instance, aims at meeting these expectations, since the search for a perfect body is still the goal of many people [2]. For increasing the volume and lifting of the buttocks, plastic surgeons have been using silicone implants for several years [3]. The use of the prosthesis helps to correct certain types of hypoplasia, and shape the glutaeus. However, depending on the anatomical plane used, and on the way the implant is positioned, there may be little satisfactory results [4]. Therefore, the anatomy of each patient should be analysed so that satisfactory long-term results can be obtained. Thus, glutaeal areas that present excesses or deficiencies should be previously identified in order to satisfy the patient [5]. Seroma formation is one of the complications resulting from this technique, which can be treated with local aspiration [6,7]. Other complications, such as asymmetry, implant migration, capsular contracture, and infection may also occur, and implant removal may be required for these cases [8]. Advances in technique, and implant options can help reduce the complication rate. Although glutaeal prostheses are widely used, there are few studies that determine their complication rates, efficacy, and safety [2]. Gluteal bioplasty is one of the techniques that has been used. With this procedure it is possible to correct asymmetry of lost implants, to treat acquired or congenital deformities, traumas or prosthesis infections, and to fill in some irregularity caused by the prosthesis itself [9]. A polymethyl methacrylate (PMMA) filler is used because it is a permanent product, stimulates collagen, and improves skin quality. The implantation procedure is also minimally invasive, and satisfactory results can be obtained.

The objective of this study is to report the case of a patient with glutaeal prostheses who was submitted to a prosthesis removal surgery, and, subsequently, to PMMA filling for correction purposes.

## Case report

**Figure 1.**
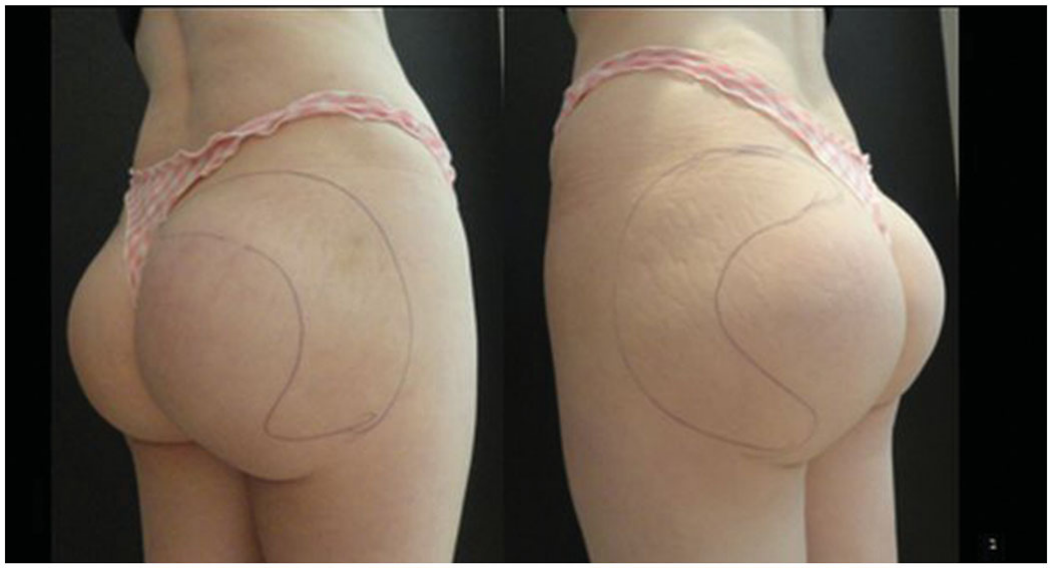
Patient with glutaeal prosthesis before treatment.

Patient D.C., female, 34 years of age, from São Paulo, went to the Clinic having glutaeal prostheses inserted using a subcutaneous technique. She reported aesthetic dissatisfaction since the prosthesis implantation. She reported she had already undergone two surgical procedures to place the prosthesis without success. During the first one, 200 mL silicone prostheses were used on each side. As the skin detachment was larger than the prostheses, the patient felt that it moved at her slightest effort. In an attempt to correct it, after two years, the patient underwent a second surgical procedure. At that time, 360 mL prostheses were inserted on each side (Figure 1) to compensate for the displaced tissue. She sought the clinic after four years of the second surgical procedure, being still dissatisfied with the results.

This case report was submitted to Plataforma Brasil (an online system run by the Brazilian federal government), and approved by the Research Ethics Committee of the Moinhos de Vento Hospital (HMV) (CAAE protocol number 25313613.4.0000.5330).

## Treatment

The treatment adopted was surgery for the removal of the prostheses, and subsequent filling of irregularities and volume with PMMA. Gluteal filling with PMMA (Linea Safe® 30%) was performed under local anaesthesia, with the patient awake accompanying by watching the results through a mirror, and actively participating of the decisions. The anaesthetic and product infiltrations were performed with a 1 mm atraumatic blunt-tipped microcanula, which causes no vasculo-nervous lesions in the glutaeal muscles, and no permanent scarring.

**Figure 2.**
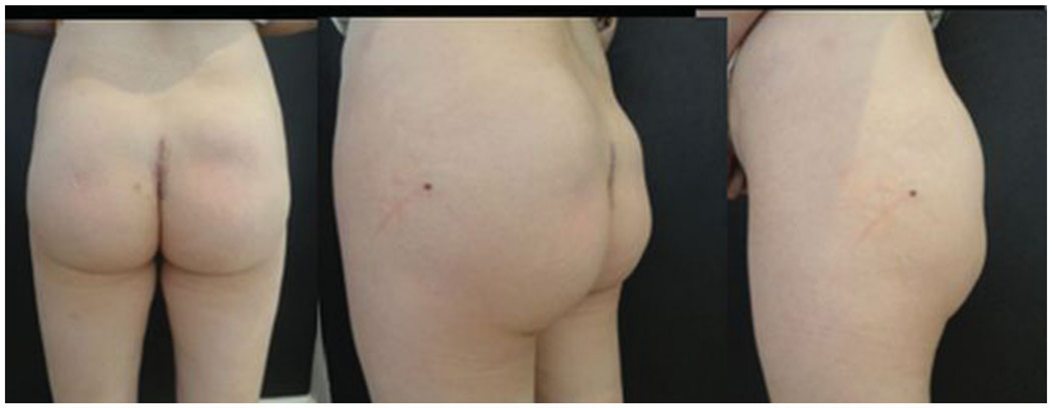
Patient after the removal of the prostheses.

After the removal of the prostheses (Figure 2) (without capsule removal), a Magnetic Resonance was done revealing a seroma which was aspirate by needle. After 3 months of prostheses removal we initiate glutaeal filling session.

In the first moment, on 02/19/2013, 90 mL of 30% PPMA were introduced in each side of the glutaeus medius and maximus muscles, and on 02/28/2013 underwent a filling session with 30% polymethyl methacrylate (adding 90 mL to each side) in the lateral side to correct the depression caused by the prosthesis. A thirty session was carried out on 09/04/2013, during which 100.5 mL of 30% PMMA were inserted in each side. On 07/07/2013, another session inserted 60 mL of 30% PMMA in each side.

**Figure 3.**
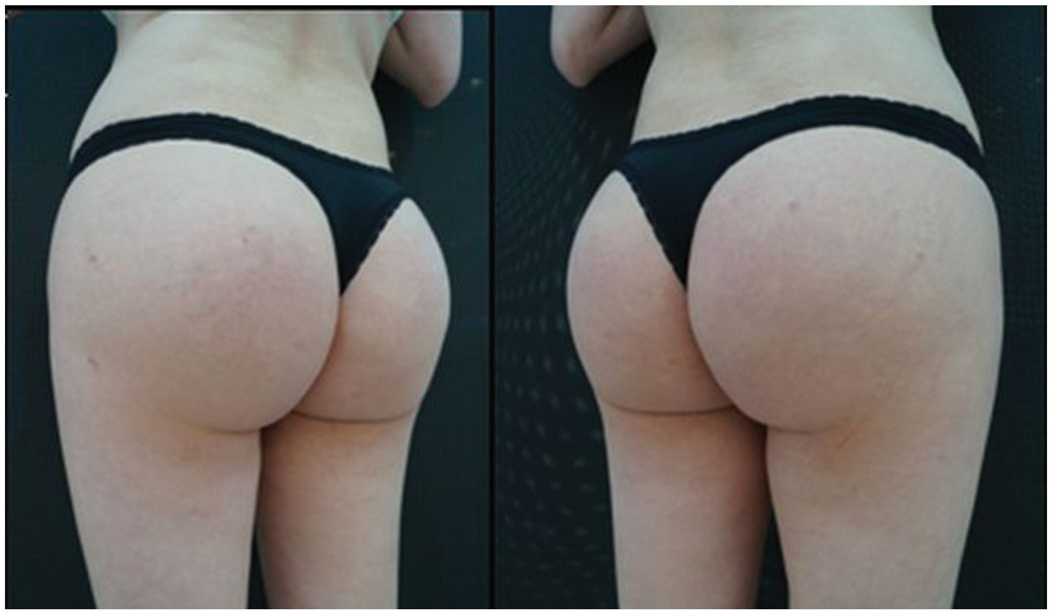
Patient after the removal of the prostheses, and PMMA filling.

After this period, 30 mL of 30% polymethylmethacrylate was introduced in each side on 08/8/2013, and 09/17/2013, totalling 60 mL in each side of the glutaeus. Thus, a satisfactory result was obtained (Figure 3). Thus, totalling 801 mL of total volume (400.5 mL on each side). At the present time (Aug/2018) no complications was reported after 5 years of follow-up.

## Discussion

Most of the buttock augmentation procedures are silicone implant surgeries, which present risks inherent to the technique and to the type of surgical approach, which can be associated with skin flap, liposculpture, and implant placement techniques.

Plastic surgery for improving body contour of the glutaeal region are increasingly sought. Badin and Vieira have described a surgical technique for the placement of high-cohesive round silicone implants using video assistance [10]. Moreover, Jaimovich et al. [11] have described anchoring sutures, and Sozer et al. have described the use of musculocutaneous flap to increase the buttock in the middle portion, and to decrease fat necrosis [12].

In an attempt to find an ideal surgical technique, Serra et al. have described easily identifiable anatomical landmarks that may assist the surgeon in performing gluteoplasty [13].

There are still few studies that determine complication rates, efficacy, and safety of using glutaeal silicone implants [2]. Post-surgical follow-up studies are required to verify the reliability of this type of prosthesis. Ford et al. [14] reported, in one paper, traumatic silicone implant rupture, migration, and granuloma formation nine years following the procedure. Early treatment using prosthesis removal surgery, when there is rupture and extravasation, is necessary. However, other alternative methods to correct irregularities presented after the removal of the prostheses are necessary. New glutaeal prostheses would be a form of repair; nonetheless, there are still the same risks of complications [14]. In another study by Aboudib et al. [15], complications, such as seroma and infection, occurred after the placement of the glutaeal prostheses, thus, requiring removal of the implants and subsequent repair.

A study by Vergara et al. [16] presented 160 patients with silicone buttock implants. Thirty patients (18.7%) had implants of 250 cc, 100 (62.5%) received 300 cc implants, and 30 (18.5%) were implanted with 350 cc silicone prostheses. There were 16 patients (10%) who presented complications, including seroma in 7 (4%), asymmetry in 4 (2.66%), capsular contracture in 3 (2%), hypercorrection in 1 (0.66%), and rupture of the implant in 1 patient (0.66%).

Cárdenas-Camarena et al. studied 62 females, and 4 males who underwent gluteoplasties in 14 years. Liposuction and lipoinjection were combined. In all cases, liposuction was also performed in other areas [17]. The infiltrated fat varied from 120 to 280 mL per glutaeus muscle, with a mean of 210 mL Follow-up ranged from 3 months to 3 years and 5 months, with an average of 17 months. Four seromas, six visible irregularities, and two palpable irregularities were seen among the cases. The complications of lipoinjection occurred in 16 glutaeus muscles (12%); all presented temporary hyperaemia and erythema, which was treated with conservative treatments, except in one case that was related to fat necrosis. A probable case of fat embolism syndrome evolved satisfactorily.

Cárdenas-Camarenas et al. [17] have studied the cases of 789 patients who underwent glutaeal liposuction and lipograft. They were injected with different volumes of fat, varying from 120 to 1160 mL. Complications, such as fat necrosis, glutaeal erythema, infection, and fat embolism syndrome were more frequent and severe in cases with smaller grafting volume.

Oranges et al. [18] performed a systematic review on the Gluteal Augmentation Techniques. They analysed historic and recent studies about negative effects on postoperative outcomes of glutaeal augmentation techniques. Oranges et al. reviewed 52 of the most important studies worldwide related to the subject. They all summed up gathered 7834 patients treated with 5 different glutaeal augmentation techniques. The authors characterised the advantages and disadvantages of each technique as follows: procedures with complications (*n* = 479) 30.5%; liposuction (*n* = 2609) with complications 10.5%; local flap (*n* = 369) complications 22%; and Hyaluronic Ac filling (*n* = 69), which present no significant complication, even though there was a smaller number of procedures performed due to the high cost and short duration of its effect [18].

Lemperle et al. [19] studied the histological reaction with several substances used for filling soft tissues. The host reacted differently to the fillers; however, all substances, being resorbable or non-resorbable, appeared to be clinically and histologically safe, even though all presented undesirable side effects

According to Chacur [20], it is possible to augment and shape the buttocks using injectable implants with various formulations. Fillers may be used in different regions of the body and face, and in each region products with different properties may be used, such as PMMA, which is used in large muscle groups

As presented in this work, after the removal of the glutaeal prostheses, the use of intramuscular fillers was an alternative for correcting acquired irregularities. The formation of neocollagen, due to the tissue stimulation that PMMA promotes, significantly improved glutaeal flaccidity. Gluteal bioplasty allows shaping and volume increasing of the buttocks. Similarly to the glutaeal prosthesis, according to studies, bioplasty is a safe technique. Gonzalez, in one study, reported that 746 patients underwent this type of procedure and obtained good results, demonstrating the safety and effectiveness of this filling technique [4].

Hilinski [21] has demonstrated improved biocompatibility as a result of increased size and uniformity of PMMA microspheres. This enhanced biocompatibility results in fewer adverse events after the placement of ArteFill thus, providing a permanent volume increase, since the non-absorbable microspheres stimulate the fibroblasts that synthesise and cause collagen deposition around them. A similar study was also conducted by Mcclelland et al [22]. The appropriate technique includes deep subcutaneous implantation, with total correction, which is gradually achieved over several treatments. Complications are limited to the formation of nodules, which are easy to handle, and, in most cases, it can be done with conservative interventions.

According to Souza et al. [23], a Brazilian consensus was reached on the use of PMMA. Their trial comprised 87,371 patients who were treated by several physicians; and 12,285 of these underwent body fillings. The overall complication index of that study was less than 1%, confirming the safety of the use of PMMA when well applied.

In this case report, in addition to the increasing in volume, which is similar to the silicone prosthesis, the aesthetic result was much better due to the possibility of PMMA distribution in regions where it is really needed, using local anaesthesia, and with the patient being awake and monitoring the result. The result was considered efficient from both the aesthetic and the technical points of view. The irregularities resulting from the removal of the prosthesis were satisfactorily corrected with the PMMA filling. No adverse effect was observed after five years of follow-up.
